# Dual inhibition of IGF-IR and ALK as an effective strategy to eradicate NPM-ALK^+^ T-cell lymphoma

**DOI:** 10.1186/s13045-019-0768-8

**Published:** 2019-07-24

**Authors:** Bhawana George, Suraj Konnath George, Wenyu Shi, Abedul Haque, Ping Shi, Ghazaleh Eskandari, Magnus Axelson, Olle Larsson, Ahmed O. Kaseb, Hesham M. Amin

**Affiliations:** 1 Department of Hematopathology, The University of Texas MD Anderson Cancer Center, Unit 072, 1515 Holcombe Boulevard, Houston, TX 77030 USA; 2 Department of Hematology, Affiliated Hospital of the University of Nantong, Jiangsu, China; 3 State Key Laboratory of Bioreactor Engineering, East China University of Science and Technology, Shanghai, China; 4 Department of Molecular Medicine and Surgery, Karolinska Institute, Stockholm, Sweden; 5 Department of Oncology and Pathology, Karolinska Institute, Stockholm, Sweden; 6 Depertment of Gastrointestinal Medical Oncology, The University of Texas MD Anderson Cancer Center, Houston, TX USA; 7 MD Anderson Cancer Center UTHealth Graduate School of Biomedical Sciences, Houston, TX USA

**Keywords:** NPM-ALK, IGF-IR, Picropodophyllin, ASP3026, T cell lymphoma, Targeted therapy

## Abstract

**Background:**

Nucleophosmin-anaplastic lymphoma kinase-expressing (NPM-ALK^+^) T cell lymphoma is an aggressive neoplasm. NPM-ALK, an oncogenic tyrosine kinase, plays a critical role in this lymphoma. Recently, selective ALK inhibitors have emerged as a first-line therapy for this neoplasm. Unfortunately, ALK inhibitors were hindered by emergence of resistance and relapse. We have previously demonstrated that type I insulin-like growth factor receptor (IGF-IR) is commonly expressed and activated in this lymphoma. In addition, IGF-IR and NPM-ALK are physically associated and reciprocally enhance their phosphorylation/activation. Herein, we tested the hypothesis that combined inhibition of IGF-IR and NPM-ALK could significantly improve the effects of inhibiting each kinase alone.

**Methods:**

We used clinically utilized inhibitors of IGF-IR (picropodophyllin; PPP) and ALK (ASP3026) to assess the in vitro cellular effects of combined treatment versus treatment using a single agent. Moreover, we used a systemic NPM-ALK^+^ T cell lymphoma mouse model to analyze the in vivo effects of PPP and ASP3026 alone or in combination.

**Results:**

Our data show that combined treatment with PPP and ASP3026 decreased the viability, proliferation, and anchorage-independent colony formation, and increased apoptosis of NPM-ALK^+^ T cell lymphoma cells in vitro. The in vitro effects of combined treatment were synergistic and significantly more pronounced than the effects of PPP or ASP3026 alone. Biochemically, simultaneous antagonism of IGF-IR and ALK induced more pronounced decrease in pIGF-IR^Y1135/1136^, pNPM-ALK^Y646^, and pSTAT3^Y705^ levels than antagonizing IGF-IR or ALK alone. Moreover, combined targeting of IGF-IR and NPM-ALK decreased significantly systemic lymphoma tumor growth and improved mice survival in vivo. Consistent with the in vitro results, the in vivo effects of the combined therapy were more pronounced than the effects of targeting IGF-IR or ALK alone.

**Conclusions:**

Combined targeting of IGF-IR and ALK is more effective than targeting IGF-IR or ALK alone in NPM-ALK^+^ T cell lymphoma. This strategy might also limit emergence of resistance to high doses of ALK inhibitors. Therefore, it could represent a successful therapeutic approach to eradicate this aggressive lymphoma. Importantly, combined inhibition is feasible because of the clinical availability of IGF-IR and ALK inhibitors. Our findings are applicable to other types of cancer where IGF-IR and ALK are simultaneously expressed.

**Electronic supplementary material:**

The online version of this article (10.1186/s13045-019-0768-8) contains supplementary material, which is available to authorized users.

## Background

Anaplastic lymphoma kinase-expressing (ALK^+^) T cell lymphoma is an aggressive neoplasm that constitutes 3–5% and 40% of adults and children/adolescents non-Hodgkin lymphoma, respectively [1]. Wild-type ALK is a receptor protein tyrosine kinase with an expression restricted to neural tissues at an early stage of human development [2]. It is believed that the phosphorylation and activation of ALK is tightly regulated through a number of yet not fully characterized ligands [3–5]. Approximately 80% of ALK^+^ T cell lymphoma cases harbor the nucleophosmin-ALK (*NPM*-*ALK*) oncogene, which results from the fusion of the *ALK* gene on the 2p23 chromosome to the *NPM* gene on the 5q35 chromosome [6]. NPM-ALK lacks an extracellular domain; however, it functions as a constitutively activated cytoplasmic tyrosine kinase that is capable of translocating to the nucleus [7]. The activation of NPM-ALK induces activation of several downstream signaling pathways including phospholipase C-γ [8], phosphoinositide 3-kinase (PI-3K)/AKT [9, 10], Janus kinases/signal transducers and activators of transcription (JAK/STAT) [11–13], and Ras/mitogen-activated protein kinase/extracellular signal-regulated kinase (MAPK/ERK) [14], all of which are crucial for cell survival and proliferation.

Treatment options for NPM-ALK^+^ T cell lymphoma were limited to conventional polychemotherapy such as the CHOP (cyclophosphamide, doxorubicin, vincristine, and prednisone) regimen. The discovery of the selective, small-molecule ALK inhibitors revolutionized the approach to treat ALK^+^ tumors. Crizitonib (PF-2341066) and NVP-TAE684 are two of the earlier first-generation ALK inhibitors [15, 16]. Although initial responses to selective ALK inhibitors are favorable, about 30% of the NPM-ALK^+^ T cell lymphoma cases develop resistance and have multiple relapses leading to disease-related morbidities and mortalities [17]. In an attempt to avoid this outcome, newer generations of ALK inhibitors were developed. Unfortunately, resistance, relapse, and disease progression also occurred when these inhibitors were used [18–20].

We have previously demonstrated that NPM-ALK is physically associated and reciprocally interacts with IGF-IR; another protein tyrosine kinase with potent oncogenic potential [21, 22]. This functional relationship appears to enhance the phosphorylation/activation of the two kinases and potentiates their effects on common downstream survival signaling JAK/STAT [12, 23]. We also found ASP3026, a second-generation ALK inhibitor that has been recently utilized in patients with ALK^+^ cancers, capable of overcoming the resistance to crizotinib-induced ALK mutants [24–26]. Notably, development of resistance to ALK inhibition has also been noted in the case of the ASP3026 small molecule inhibitor [27]. A previous study also reported that IGF-IR constitutive activation could be an important factor contributing to the acquired resistance to ALK inhibitors [28]. Collectively, these data warrant the search for more refined strategies to overcome these hurdles, and suggest that it is still possible that the utilization of significantly smaller doses of ALK inhibitors in combination with other legitimate targeted therapies, such as IGF-IR inhibitors, may limit significantly the resistance to higher doses of ALK when used alone.

Picropodophyllin (PPP; AXL1717) is a clinically utilized, selective, small molecule inhibitor of IGF-IR [29–33]. It has been shown to be effective in inhibiting various types of cancers including those of the gastrointestinal tract, nasopharynx, liver, lung, ovary, soft tissues, and hematopoietic system including NPM-ALK^+^ T cell lymphoma [21, 34–46]. Recently, however, higher doses of PPP have been shown to induce bone marrow toxicity in some patients [30]. In this paper, we tested the hypothesis that dual suppression of ALK and IGF-IR could represent a superior strategy that significantly improves the effects of the isolated inhibition of each enzyme alone. It is also anticipated that this approach might lead to decreased acquired resistance and eliminate potential unwarranted side effects that may associate the utilization of higher doses.

To achieve our goals, we used low doses of ASP3026 and PPP, and compared their in vitro and in vivo effects alone or combination. Our in vitro data demonstrated that low doses of ASP3026 in combination with PPP act synergistically to exert more pronounced anti-proliferative and apoptotic effects on NPM-ALK^+^ T cell lymphoma cells than the effects of each drug alone. In a systemic NPM-ALK^+^ T cell lymphoma mouse model, combined treatment with ASP3026 and PPP was associated with slower tumor growth and longer survival when compared with individual drug treatments. Considering that the two inhibitors have been individually utilized in patients, our data stress the feasibility of the combination strategy to be further tested in the clinic.

## Methods

### IGF-IR and ALK inhibitors

PPP was dissolved in DMSO for in vitro studies, and in DMSO/vegetable oil (10:1) for in vivo studies. ASP3026 (CT-ASP302; ChemieTek, Indianapolis, IN) was dissolved in DMSO with H_2_O and HCl (1:1) *for in vitro* experiments, and in 0.5% methyl cellulose for in vivo experiments.

### Cell lines

The NPM-ALK^+^ T cell lymphoma cell lines Karpas 299, DEL, and SR-786 were purchased from DSMZ (Braunschweig, Germany). Cells were maintained in RPMI 1640 plus 10% FBS, 2 mM glutamine, 100 U/mL penicillin, and 100 μg/mL streptomycin, in a humidified chamber at 37 °C with 5% CO_2_.

### Generation of cells resistant to the ALK inhibitor ASP3026

DEL cells were cultured in increasing concentrations of ASP3026 (0.125–1.0 μM) for 4 months. Acquired resistance to 1.0 μM ASP3026 (DEL-R cells) was determined by an MTS assay as described below.

### Antibodies

The following antibodies were used for Western blotting: pALK^Y1586^ (NPM-ALK^Y646^; catalogue number: 3343), IGF-IR (9750), pIGF-IR^Y1135/1136^ (3024), STAT3 (9139), pSTAT3^Y705^ (4113), PARP (9532), cleaved PARP (5625) (Cell Signaling, Danvers, MA), ALK (M719501-2) (Dako, Carpinteria, CA), β-actin (A-2228) (Sigma-Aldrich, St. Louis, MO). Antibodies used for immunohistochemical staining are described below.

### MTS assay

Cell viability was measured using the MTS reagent 3-(4,5-dimethylthiazol-2-yl)-5-(3carboxymethoxyphenyl)-2-(4-sulfophenyl)-2H-tetrazolium (Promega, Madison, WI). Briefly, cells (1 × 10^6^/mL) were seeded into 96-well plates and treated with relevant drugs after overnight incubation. Thereafter, each well was incubated with 20 μL of MTS reagent for 1 to 4 h at 37 °C in 5% CO_2_. Absorbance at 490 nm was read using a plate reader.

### Isobolographic analysis

We used the concentration-dependent viability (MTS) assay curves to generate isoeffect curves by using isobolograms to determine whether the net effects of PPP and ASP3026 were synergistic, additive, or antagonistic. Concentration-dependent effects were calculated using Excel software (Microsoft, Redmond, WA) for one drug while keeping constant concentrations for the other.

### Apoptosis detection

Induction of apoptosis was detected by annexin V-FITC and PI-based flow cytometric analysis using apoptosis detection kit (556547, BD Biosciences). Briefly, cells were simultaneously stained with annexin V-FITC and PI and incubated for 15 min at room temperature in 1× annexin binding buffer. Fluorescence intensity was measured by flow cytometry (BD FACSCalibur system). This assay distinguishes cells that are intact (FITC^−^/PI^−^), apoptotic (FITC^+^/PI^−^), or necrotic (FITC^+^/PI^+^). Analysis was performed using flow cytometry (BD FACSCalibur, BD Biosciences, San Jose, CA). The percentage of cells undergoing apoptosis was quantified by CellQuest software (BD Biosciences).

### Cell proliferation

Cell proliferation was measured by a BrdU assay using a standard kit (X1327K1, Exalpha Biologicals, Shirley, MD). Cells were plated at a concentration of 2 × 10^5^ cells/well. Thereafter, 20 μL of BrdU (1:500) were added for 24 h. Plates were centrifuged for 30 min, and 100 μL of anti-BrdU antibody were added for 1 h, followed by 100 μL peroxidase goat anti-mouse IgG conjugate (1:2000) for 30 min. Thereafter, 100 μL of TMB substrate were added for 30 min. Stop solution (50 μL) was added and plates were read at 450/595 nm.

### Anchorage independent colony formation

Cells were plated in a methylcellulose-based medium (Methocult H4230; Stemcell Technologies, Vancouver, BC, Canada) mixed in RPMI-1640 (1:4). Harvested cells were mixed in a 1:10 (*v*/*v*) ratio with methylcellulose media in a 15 mL conical vial. Tubes were inverted to mix the contents and poured to 6-well plates without any bubbles. The plates were then incubated at 37 °C in a 5% CO_2_ incubator for approximately 5 days. In order to stain the colonies, p-iodonitrotetrazolium violet was added and incubated for 24 h. Colonies were visualized using the FluorChem 8800 imaging system (Alpha Innotech, San Leandro, CA).

### Western blotting

Lysis buffer contained 25 mM HEPES (pH 7.7), 400 mM NaCl, 1.5 mM MgCl_2_, 2 mM EDTA, 0.5% Triton X-100, 0.1 mM phenylmethylsulfonyl fluoride, 3 mM dithiothreitol, phosphatase inhibitor cocktail (20 mM β-glycerol phosphate, 1.0 mM sodium orthovanadate), and protease inhibitor cocktail (10 μg/mL leupeptin, 2 μg/mL pepstatin, 50 μg/mL antipain, 1× benzamidine, 2 μg/mL aprotinin, 20 μg/mL chymostatin). Further, 50 μg total proteins were electrophoresed on 10% SDS-PAGE, transferred onto nitrocellulose membranes, and probed with primary antibodies, followed by matched secondary antibodies conjugated with horseradish peroxidase (HRP). Protein expression was detected using chemiluminescence and a commercially available kit (GE Healthcare, Piscataway, NJ).

### Systemic NPM-ALK^+^ T cell lymphoma mouse model

In vivo studies were approved by the Institutional Animal Care and Use Committee. Karpas 299 cells permanently expressing firefly luciferase and GFP were generated by using the F-Luc-GFP lentivirus (Capital Biosciences, Rockville, MD) in which humanized firefly luciferase (hLUC) was expressed under the CMV promoter, and GFP with puromycin resistance marker were co-expressed bicistronically under the SV40 promoter. C.B-17 SCID mice (6–8-week-old females) were purchased from Taconic Biosciences (Cambridge City, IN). Moreover, 1.0 × 10^6^ cells were injected via tail vein and allowed to establish disseminated malignant lymphoma for about 3 weeks. To monitor lymphoma, d-luciferin (Gold Biotechnology, St. Louis, MO) was i.p. injected and luciferase signaling was detected using bioluminescence imaging (IVIS Lumina XR imaging system; Caliper Life Sciences, Alameda, CA). Starting from the third week, a low dose of ASP3026 (5 mg/kg/day) was administered once a day by oral gavage for 6 weeks. PPP (20 mg/kg) was administered i.p. every 12 h only for 3 weeks. In a subgroup of mice, CHOP was administered at 1/3 of the standard dose (cyclophosphamide, 13.3 mg/kg; doxorubicin, 1.1 mg/kg, and vincristine, 0.166 mg/kg, were all administered intravenously; whereas prednisone, 0.06 mg/kg, was given once a day by using oral gavage) for five consecutive days at the beginning of the third week. Tumor progression was monitored weekly until death or study conclusion. Kaplan–Meier survival curves were used to determine the efficacy of individual or combined drugs compared to controls. Mice underwent total necropsy, and tumors were fixed in 10% buffered formalin or snap-frozen in liquid nitrogen.

### Immunohistochemical staining

Lymphoma tumors from mice were processed by fixation in formalin followed by paraffin embedding. Prior to staining, tumors were deparaffinized in alcohol gradient, washed, and then subjected to antigen retrieval for 45 min in a steamer using Target Retrieval Solution (1×; pH 9.0, Dako). Sections were left for 20 min to cool down to room temperature, washed, and incubated in 3% H_2_O_2_ for 15 min to block endogenous peroxidase activity. Sections were then blocked for 30 min at room temperature in serum-free blocking solution (Dako). The primary antibody, diluted in blocking buffer, was added for overnight incubations at 4 °C. Primary antibody dilutions were 1:50 for ALK (M719501-2, Dako) and pSTAT3^Y705^ (4113, Cell Signaling); and 1:75 for pIGF-IR^Y1161^ (ab39398, Abcam, Cambridge, MA) and Ki-67 (M7240, Dako). Sections were washed three times and incubated for 30 min with the secondary antibody Dako Envision+Link System-HRP. Signals were developed using 3,3’-diaminobenzidine tetrachloride substrate, and hematoxylin was used for counterstaining.

### Statistical analysis

In vitro assays were set up in triplicates and the results were expressed as means ± S.D. Statistical significance between the experimental groups were detected by using student’s *t* test or two-way analysis of variance (ANOVA), where appropriate. All statistical analyses including generation of the Kaplan–Meier curves were performed by using the GraphPad PRISM software (GraphPad Software Inc., San Diego, CA). *P* < 0.05 was considered statistically significant.

## Results

### Combined treatment with PPP and ASP3026 reduces effectively the viability of NPM-ALK^+^ T cell lymphoma cells in a concentration-dependent manner

**Fig. 1:**
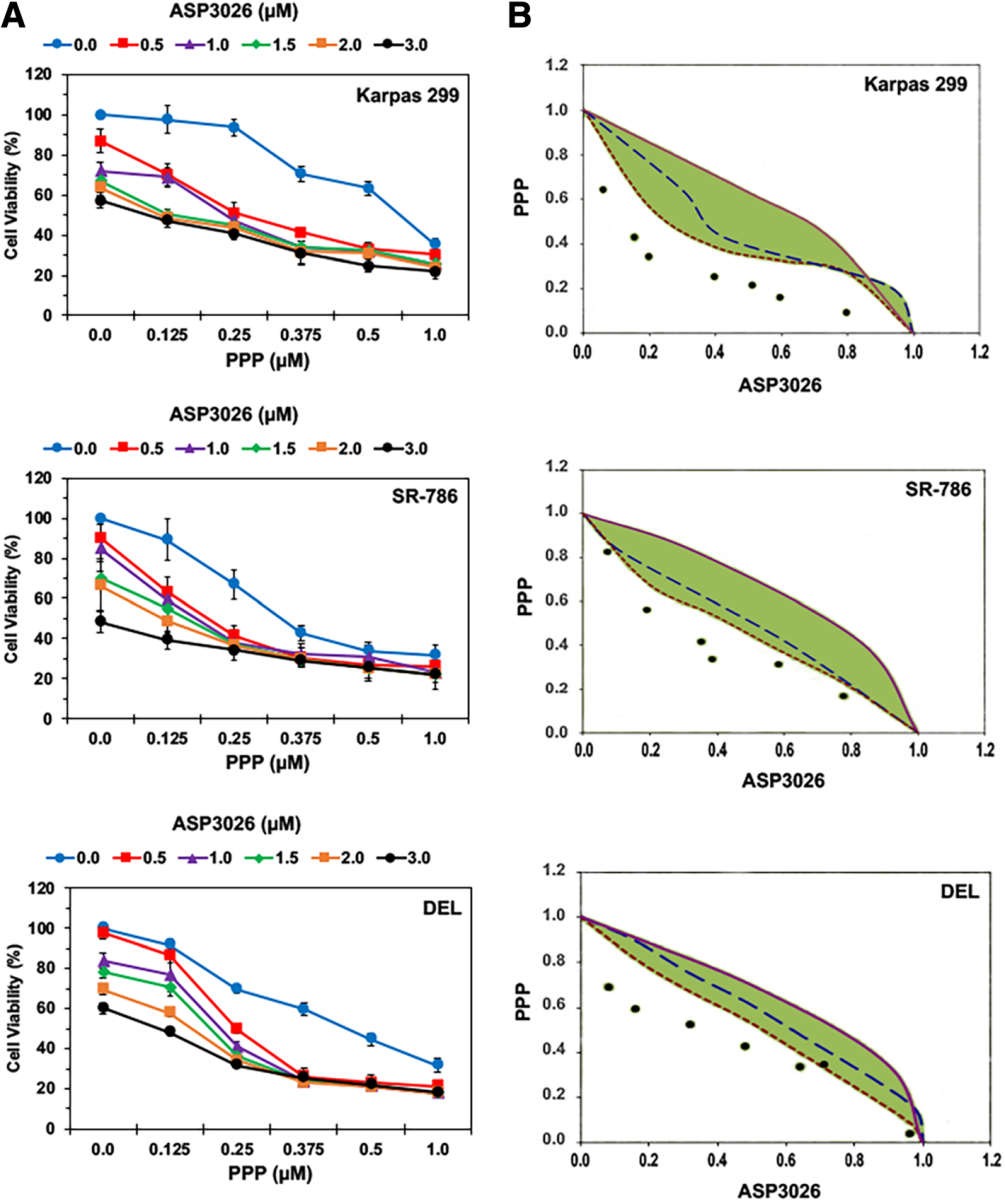
Combined treatment with PPP and ASP3026 decreases the viability of NPM-ALK^+^ T cell lymphoma cells. **a** The combination of PPP and ASP3026 decreased the viability of Karpas 299, SR-786, and DEL cells to a much greater extent than when each of the two drugs was used alone. Results are shown as means ± SD of three independent experiments. **b** Isobolographic curves of Karpas 299, SR786, and DEL cells show that the data points lie to the left of the envelope of additivity (green area), supporting a synergistic effect of the combination treatment using PPP and ASP3026

Karpas 299, SR-786, and DEL cells were treated with PPP and ASP3026 and cell viability was measured after 48 h by using an MTS assay. PPP or ASP3026 alone reduced lymphoma cell viability in a concentration-dependent manner. However, the combination of PPP and ASP3026 reduced cell viability to a much greater extent than either of these two drugs alone (*P* < 0.0001 for combination vs. PPP or ASP3026 alone in the three cell lines; Fig. 1a). To assess whether the combined effects of PPP and ASP3026 were additive or synergistic, isobologram analysis was performed. The area enclosed by the isoeffect curves is referred to as the envelope of additivity. When the data points of the drug combination fall within the envelope, the combined effects are considered additive. Deviation of data points to the left of the envelope indicate that the combined effects are caused by low concentrations of the two agents than is predicted, and the combination effect is regarded as synergistic [47]. Isobologram analysis of Karpas 299, SR-786, and DEL cells showed that the data points are located to the left of the envelope, indicating synergistic interactions between PPP and ASP3026 (Fig. 1b).

### Dual treatment with PPP and ASP3026 decreases significantly the proliferation of NPM-ALK^+^ T cell lymphoma cells

**Fig. 2:**
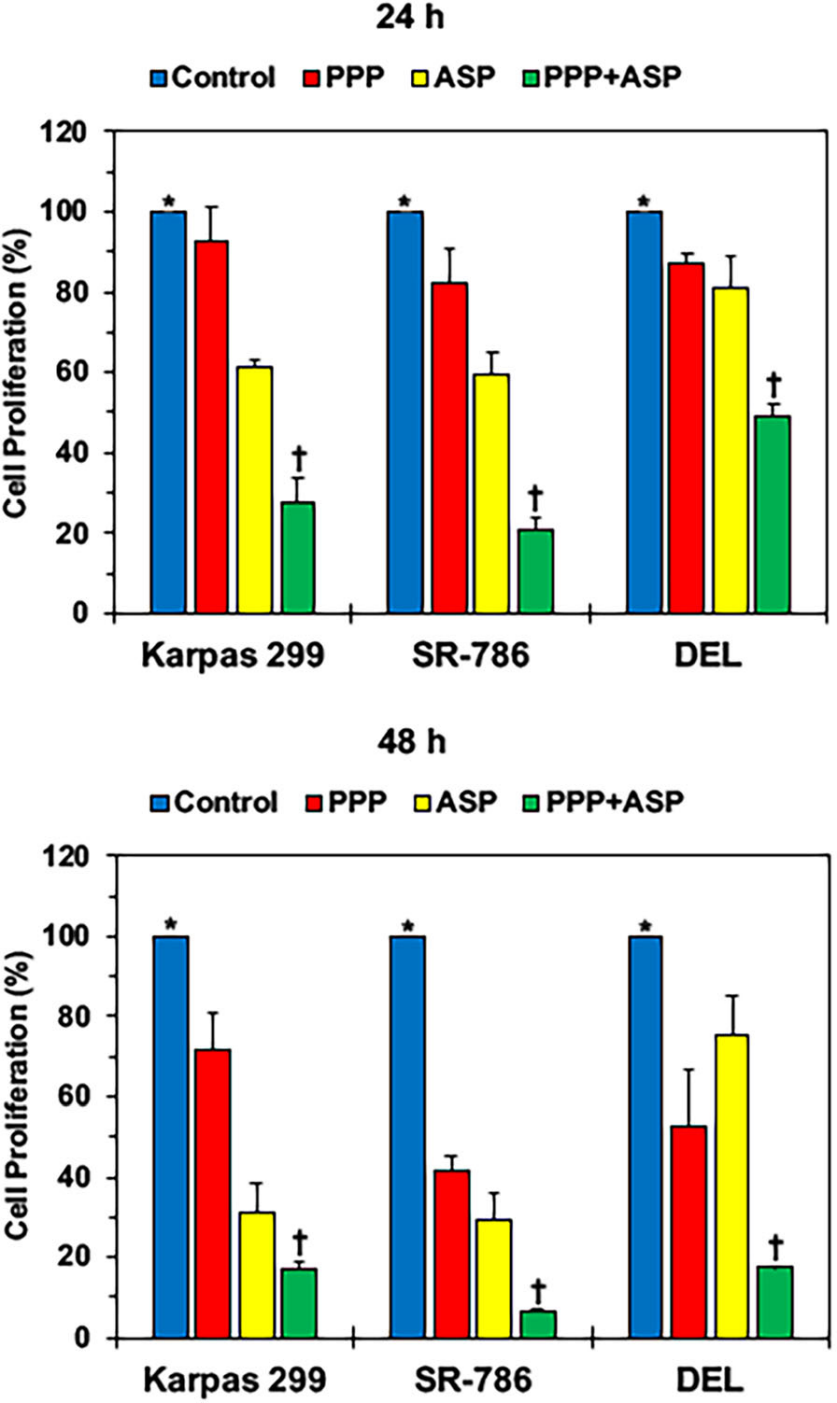
Combined treatment with PPP and ASP3026 decreases significantly the proliferation of NPM-ALK^+^ T cell lymphoma cells. Compared with control untreated lymphoma cells, treatment with a low concentration of PPP (0.25 μM) or ASP3026 (1.0 μM) alone for 24 h decreased cell proliferation in Karpas 299, SR-786, and DEL cells. Importantly, the combination treatment decreased cell proliferation more significantly than the effects of a single agent alone. Similar effects, yet more pronounced, were noted after 48 h. Results are shown as means ± SD of three independent experiments

To further probe whether combining PPP with ASP3026 enhances the effects of each of the two drugs, changes in cell proliferation were measured using a BrdU assay. At 24 h, a low concentration of PPP (0.25 μM) decreased the proliferation of SR-786 and DEL cells. In addition, ASP3026 (1.0 μM) decreased the proliferation of the three cell lines (Fig. 2, upper panel). Of note, the combination of PPP and ASP3026 suppressed cell proliferation much more efficiently than ASP3026 or PPP alone. Compared with control untreated lymphoma cells, treatment with a low concentration of PPP (0.25 μM) or ASP3026 (1.0 μM) alone for 24 h decreased cell proliferation in Karpas 299 (*: *P* < 0.0001 for control vs. ASP3026); SR-786 (*: *P* < 0.05 vs. PPP; *P* < 0.001 vs. ASP3026); and DEL (*: *P* < 0.01 vs. PPP; *P* < 0.05 vs. ASP3026). Importantly, the combination treatment decreased cell proliferation more significantly than the effects of a single agent alone (†: *P* < 0.0001 for combination vs. control, PPP or ASP3026). Figure 2, lower panel, shows that similar effects, yet more pronounced, were noted after 48 h. Compared with control cells, treatment of with PPP or ASP3026 alone decreased cell proliferation in Karpas 299 (*: *P* < 0.01 vs. PPP; *P* < 0.0001 vs. ASP3026); SR-786 (*: *P* < 0.0001 vs. PPP and ASP3026); and DEL (*: *P* < 0.01 vs. PPP; *P* < 0.05 vs. ASP3026). The combination of PPP and ASP3026 suppressed cell proliferation efficiently as well (*: *P* < 0.0001 for control vs. combination). Notably, the combination regimen reduced cell proliferation more significantly than PPP or ASP3026 alone in Karpas 299 (†: *P* < 0.001 vs. PPP; *P* < 0.05 for vs. ASP3026); SR-786 (†: *P* < 0.001 vs. PPP; *P* < 0.01 vs. ASP3026); and DEL (†: *P* < 0.05 vs. PPP; *P* < 0.001 vs. ASP3026).

### Combining PPP and ASP3026 enhances apoptotic cell death in NPM-ALK^+^ T cell lymphoma cells

**Fig. 3:**
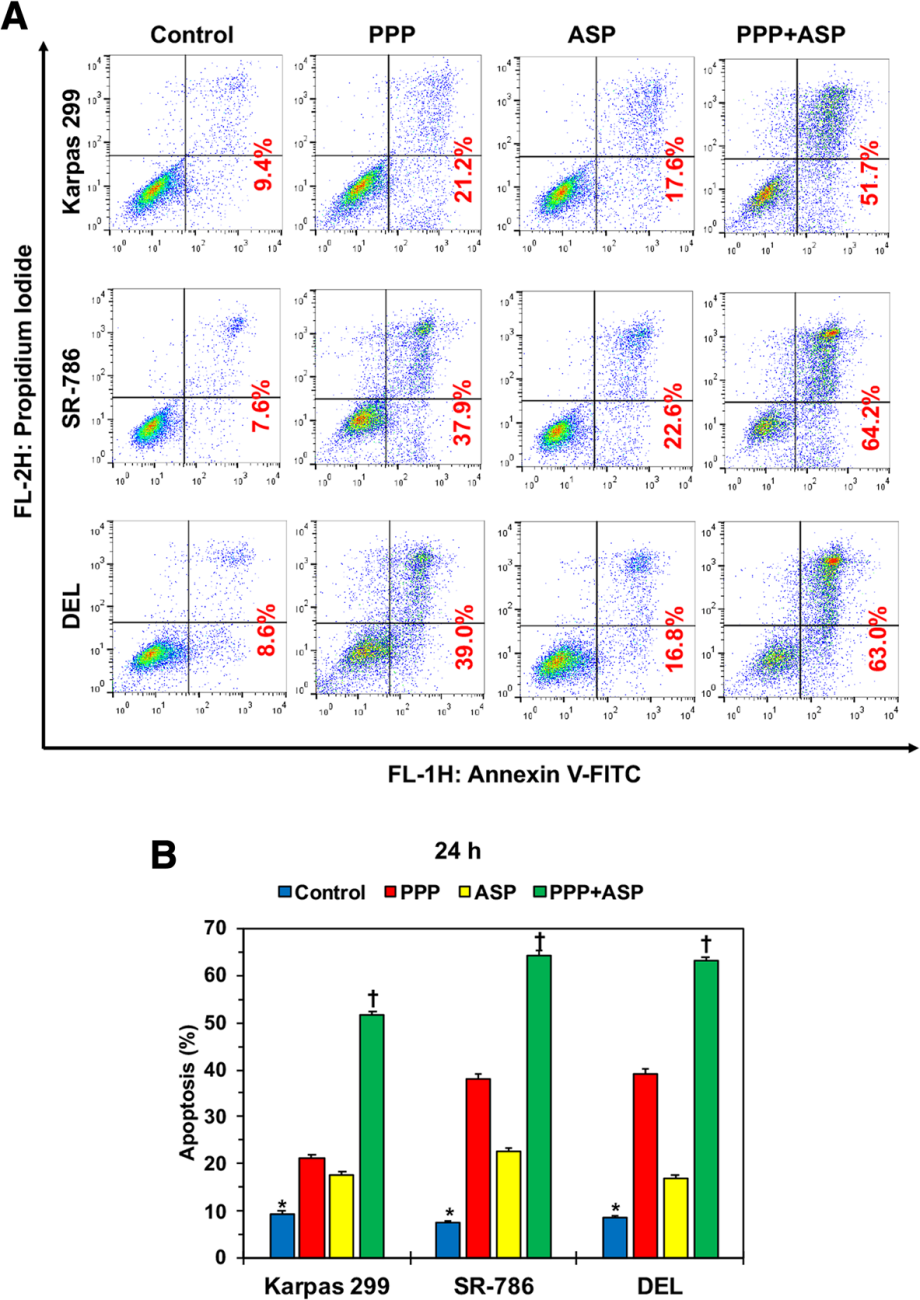
Dual treatment with PPP and ASP3026 enhances apoptosis in NPM-ALK^+^ T cell lymphoma cells. **a** Representative examples of apoptosis flow cytometry analysis charts in the three cell lines. Although treatment with PPP or ASP3026 alone was associated with increased early apoptotic (right lower quadrant) or late apoptotic + necrotic (right upper quadrant) cells, combined treatment induced a much more pronounced increase in these cellular fractions in the three cell lines. Percentage of cells undergoing early and late apoptosis are shown. **b** Compared with control cells, treatment with a low concentration of PPP (0.25 μM) or ASP3026 (1.0 μM) for 24 h was associated with apoptotic cell death in the NPM-ALK^+^ T cell lymphoma cell lines. Also, the increase in apoptosis was markedly pronounced in cells treated simultaneously with the two agents compared to control cells. However, the combination treatment increased apoptosis more significantly than treatment with PPP or ASP3026 alone. Results are shown as means ± SD of three independent experiments

Flow cytometric analysis of apoptosis was performed after treatment of the cells and dual staining with annexin V-FITC and PI. Figure 3 illustrates that, compared with control cells, treatment with a low concentration of PPP (0.25 μM) or ASP3026 (1.0 μM) for 24 h was associated with apoptotic cell death in Karpas 299 (*: *P* < 0.0001 for control vs. PPP; *P* < 0.001 vs. ASP3026); SR-786 (*: *P* < 0.0001 vs. PPP and ASP3026); and DEL (*: *P* < 0.0001 vs. PPP; *P* < 0.001 vs. ASP3026). Also, the increase in apoptosis was markedly pronounced in cells treated simultaneously with the two agents compared to control cells (*: *P* < 0.0001). Importantly, apoptosis induced by combined treatment was much more pronounced than apoptosis induced by any of the two drugs when used alone (†: *P* < 0.0001 vs. PPP or ASP3026).

### Combining PPP and ASP3026 is more superior than each drug alone in abrogating anchorage-independent colony formation of NPM-ALK^+^ T cell lymphoma cells

**Fig. 4:**
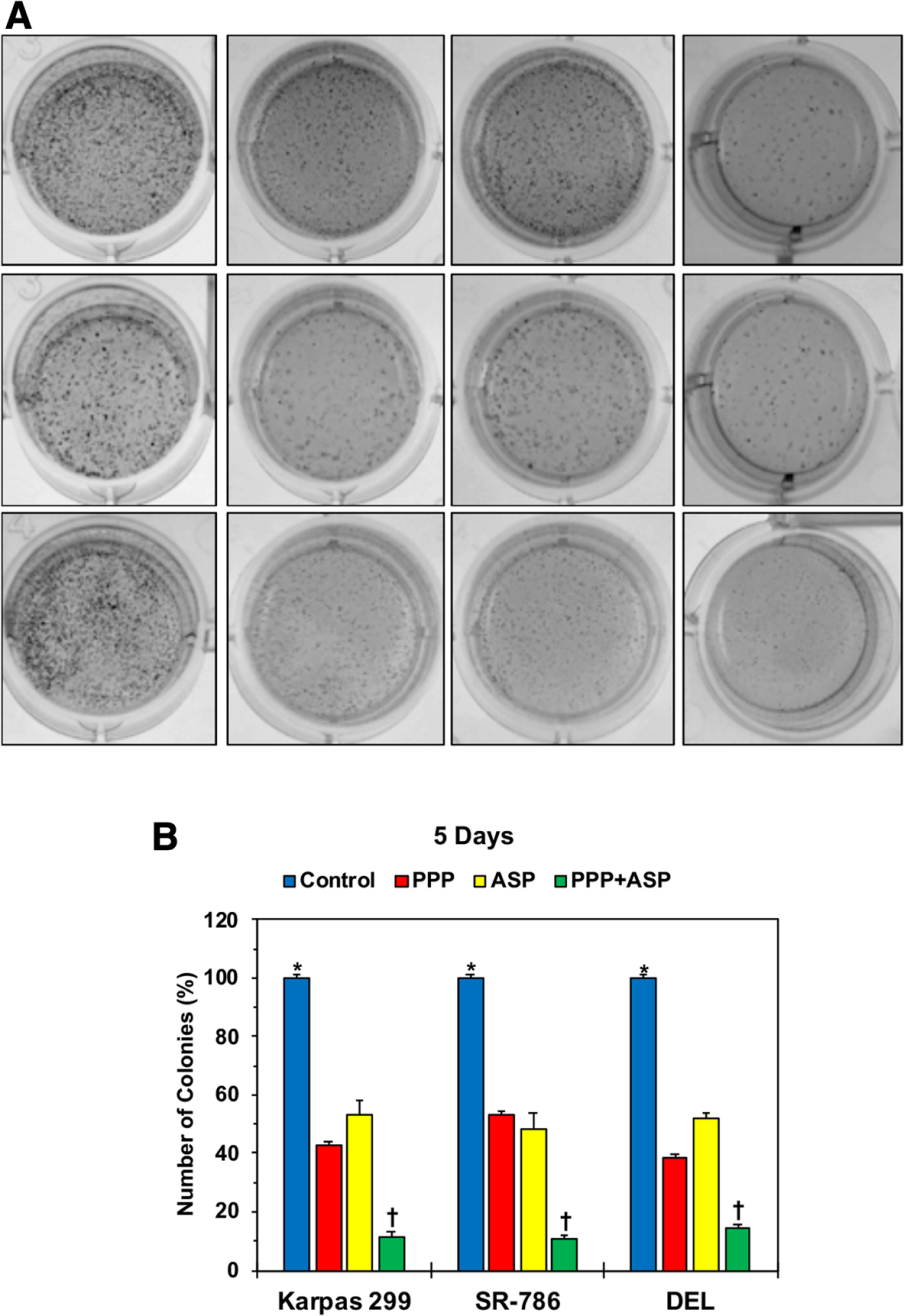
Simultaneous treatment with PPP and ASP3026 abrogates anchorage-independent colony formation of NPM-ALK^+^ T cell lymphoma cells. **a** Representative images of colonies from cells treated with ASP3026 or PPP alone and in combination are shown. **b** Treatment of the NPM-ALK^+^ T cell lymphoma cell lines with PPP (0.25 μM) or ASP3026 (1.0 μM) for 48 h was associated with a significant decrease in anchorage-independent colony formation at 5 days after treatment, and the decrease in colony formation was also markedly pronounced in cells treated with combined drugs. Importantly, combination regimen reduced colony numbers more efficiently than a single treatment with PPP or ASP3026. The results are presented as means ± SD of three independent experiments

Treatment with ASP3026 (1.0 μM) or PPP (0.25 μM) alone decreased anchorage-independent colony formation of Karpas 299, SR-786, and DEL cells at 5 days after initiating the experiment (*: *P* < 0.0001 for control vs. PPP or ASP3026). The decrease in colony formation was also markedly pronounced in cells treated with combined drugs (*: *P* < 0.0001 for control vs. combination). Importantly, combination regimen reduced colony numbers more efficiently than PPP or ASP3026 alone in Karpas 299 (‡: *P* < 0.001 vs. PPP or ASP3026); SR-786 (‡: *P* < 0.0001 vs. PPP; *P* < 0.001 vs. ASP3026); and DEL (‡: *P* < 0.0001 vs. PPP or ASP3026), (Fig. 4).

### Effects of PPP and ASP3026 on survival-promoting signaling pathways in NPM-ALK^+^ T cell lymphoma cells

**Fig. 5:**
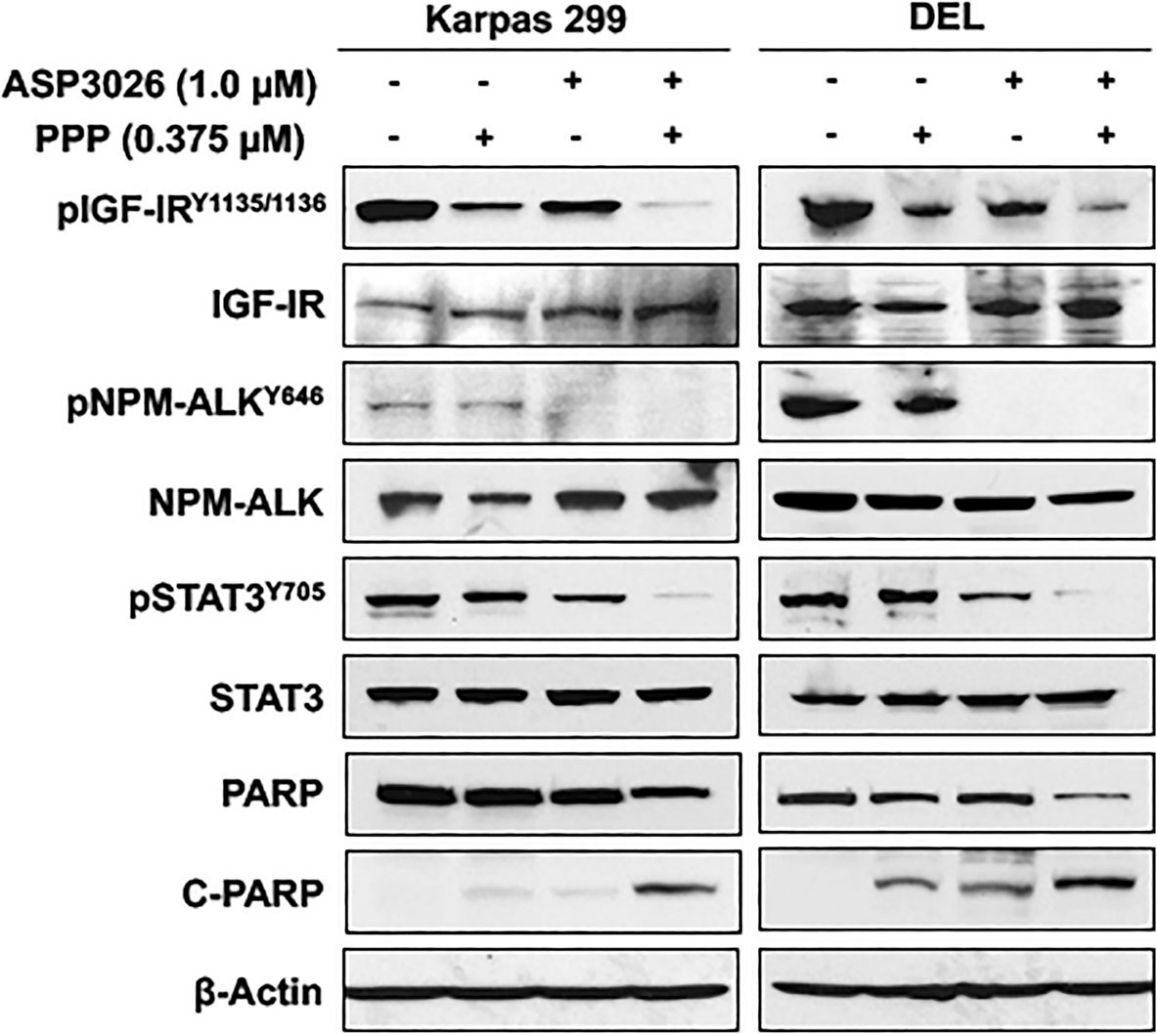
Effects of combining treatment with PPP and ASP3026 on survival proteins in NPM-ALK^+^ T cell lymphoma cells. Western blotting shows that at 48 h, treatment with a low concentration of PPP (0.375 μM) alone was associated with downregulation of IGF-IR phosphorylation in Karpas 299 and DEL cells. In addition, ASP3026 (1.0 μM) alone induced a notable decrease in the phosphorylation of NPM-ALK, IGF-IR, and STAT3. Nevertheless, the addition of the low concentration of PPP to ASP3026 induced dramatic downregulation of phosphorylated IGF-IR, NPM-ALK, and STAT3. Occurrence of apoptosis was biochemically supported by an increase in cleaved PARP (C-PARP) and a simultaneous decrease in non-cleaved PARP levels. These observations were remarkably more pronounced after the combined treatment. β-Actin indicates equal protein loading

To seek an explanation for the more pronounced effects of combination treatment with PPP and ASP3026, we measured phosphorylation levels of IGF-IR, NPM-ALK, and STAT3 in NPM-ALK^+^ T cell lymphoma cells. Whereas phosphorylated IGF-IR, NPM-ALK, and STAT3 appeared to be almost completely abrogated by PPP combined with ASP3026 in the lymphoma cells, PPP or ASP3026 alone induced significantly lesser effects on these survival-promoting proteins (Fig. 5). Moreover, combining PPP and ASP3026 was more effective in cleaving PARP than PPP or ASP3026 alone (Fig. 5), which could explain, at least in part, the enhanced apoptotic effects of combined treatment (Fig. 3).

### Acquired resistance to ALK inhibition is associated with upregulation of phosphorylated IGF-IR as well as conserved sensitivity to IGF-IR inhibition

**Fig. 6:**
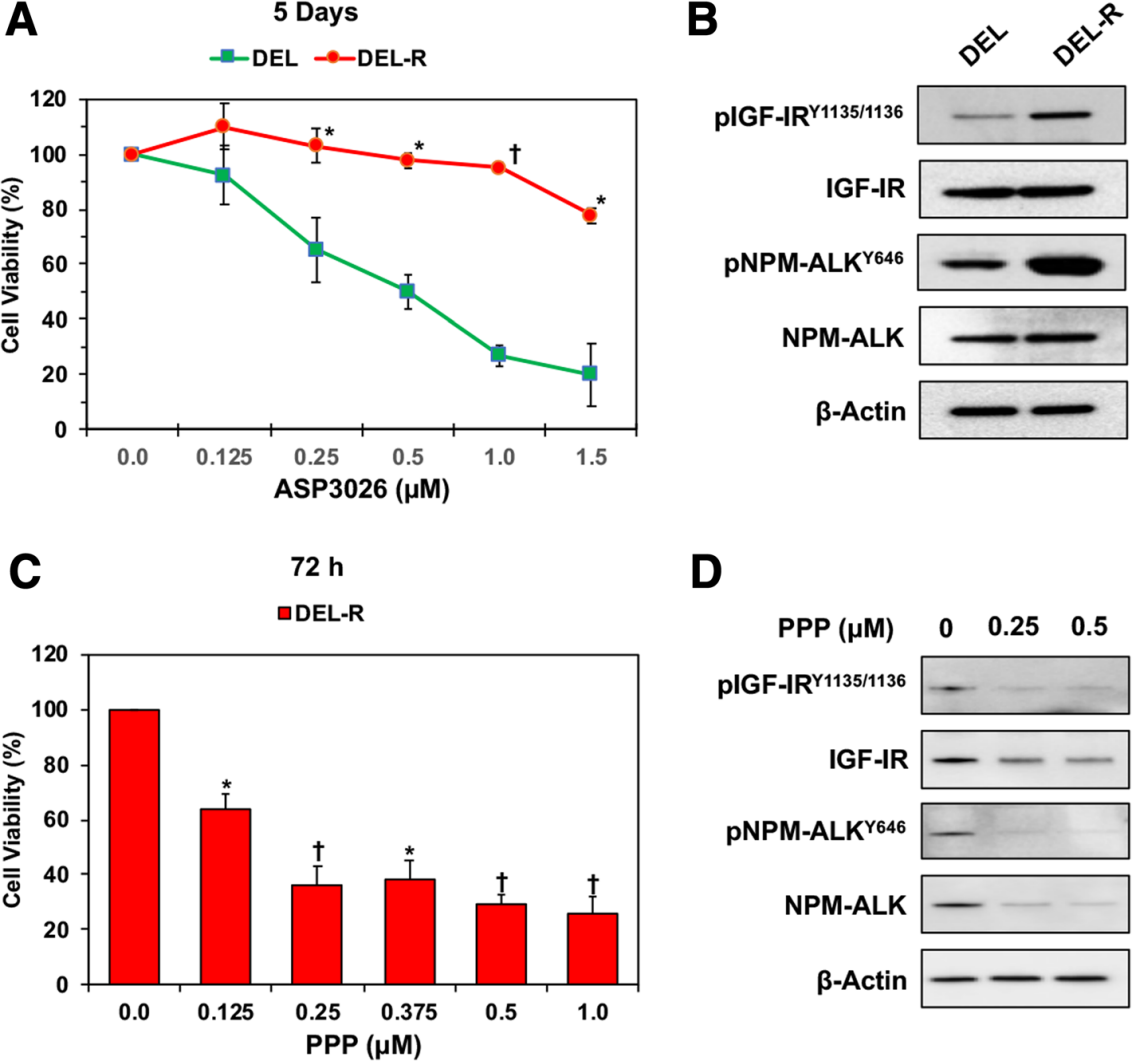
Acquired resistance to ALK inhibition by ASP3026 is associated with upregulation of IGF-IR phosphorylation. **a** DEL and DEL-R (resistant to ASP3026) cells were treated for 5 days with increased concentrations of ASP3026, and cell viability was then determined. Treatment with ASP3026 was associated with significantly more reduction in the viability of DEL cells than the viability of the DEL-R cells. **b** Western blotting shows that the DEL-R cells have upregulated phosphorylation of NPM-ALK and IGF-IR. Changes in the basal levels of these proteins were not detected. β-Actin demonstrates equal protein loading. **c** Despite that the DEL-R cells demonstrated substantial resistance to ASP3026, PPP, at 72 h, decreased effectively the viability of these cells in a concentration-dependent manner. The results are shown as means ± SD of three independent experiments. **d** Treating the DEL-R cells with PPP was associated with substantial downregulation of IGF-IR, pIGF-IR, NPM-ALK, and pNPM-ALK. β-Actin supports equal protein loading

We cultured parental DEL cells in increasing concentrations of ASP3026 over a period of 4 months. Treatment with ASP3026 led to development of a resistant clone (DEL-R) that was able to continue growing in the presence of 1.0 μM of ASP3026 while wild-type DEL cells did not survive. The effects of increasing concentrations of ASP3026 on cell viability after 5 days of treatment are shown in Fig. 6a. Treatment with ASP3026 was associated with significantly more reduction in the viability of DEL cells than the viability of DEL-R cells (*: *P* < 0.01; †: *P* < 0.001 in DEL-R vs. DEL). Similar results were obtained at shorter durations (data not shown). Interestingly, a remarkable increase not only in phosphorylated NPM-ALK but also in phosphorylated IGF-IR was observed in DEL-R cells compared to the parental DEL cells (Fig. 6b). Importantly, at 72 h after treatment, PPP was still effective in decreasing the viability of DEL-R cells despite them being resistant to ASP3026 (*: *P* < 0.001; †: *P* < 0.0001 vs. control), (Fig. 6c). The PPP-induced decrease in the viability of the DEL-R cells could be attributed, at least in part, to associated downregulation of the expression of IGF-IR, pIGF-IR, NPM-ALK, and pNPM-ALK (Fig. 6d).

### Combined treatment with low doses of PPP and ASP3026 induces robust NPM-ALK^+^ T cell lymphoma tumor suppression in vivo

**Fig. 7:**
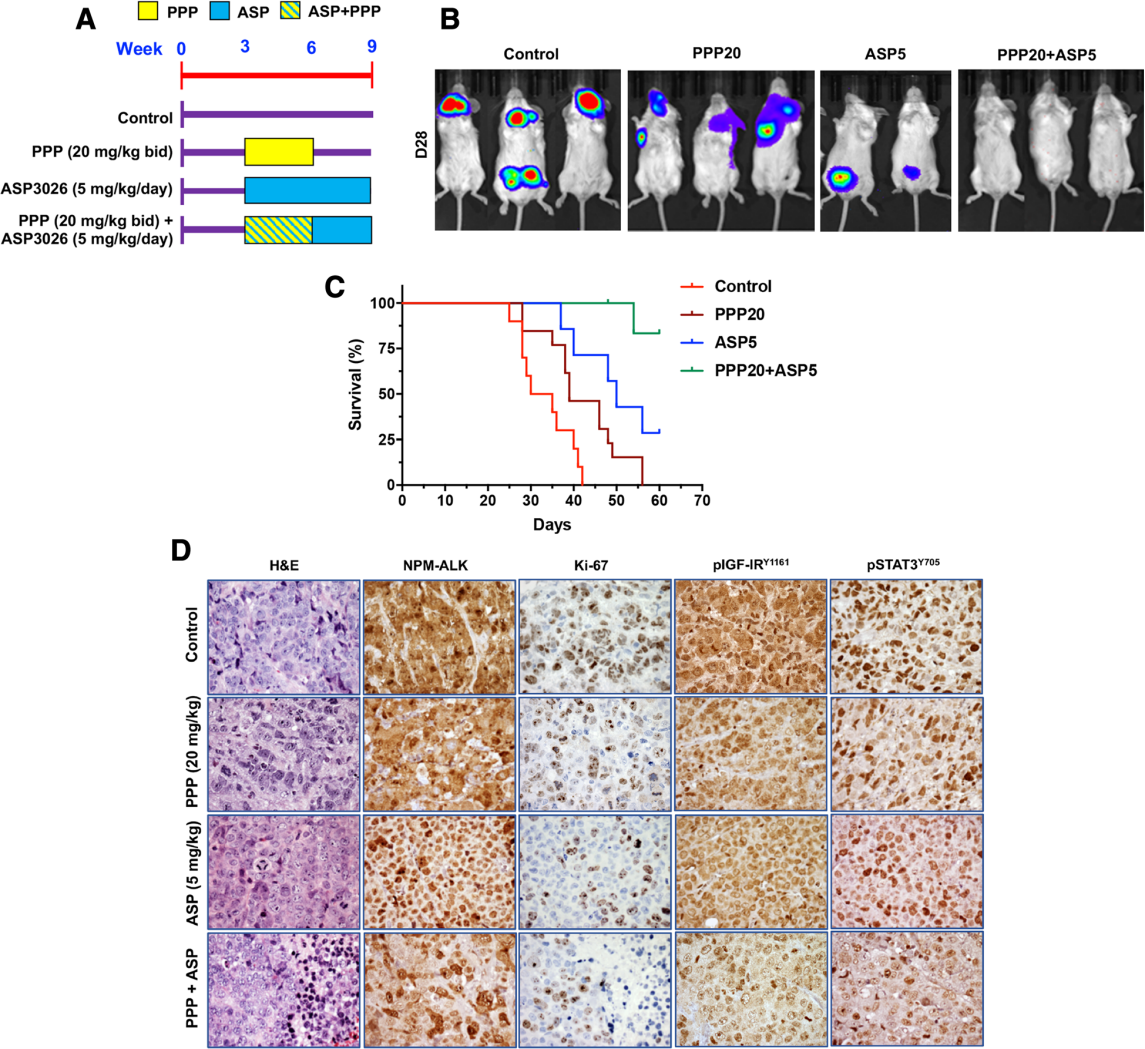
Combined treatment with PPP and ASP3026 suppresses NPM-ALK^+^ T cell lymphoma cell tumor growth in vivo*.* **a** C.B-17 SCID mice were randomized into four treatment groups at week 3 after i.v. injection of Karpas 299 cells permanently expressing firefly luciferase. Thereafter, mice were treated as illustrated. **b** Mice developed lymphoma tumors within approximately 3 weeks after injection of Karpas 299 cells, and were monitored weekly using bioluminescence imaging. The intensity of the signal is indicated by color (blue: low; green: intermediate; red: high tumor burden). Control mice treated only with vehicle showed aggressive tumor growth. Mice treated with a PPP (20 mg/kg) or a low dose of ASP3026 (5 mg/kg) alone showed reduced tumor burden compared to vehicle-treated mice. Importantly, mice that received the combination regimen showed almost complete absence of tumor growth after 4 weeks from the injection. **c** Kaplan–Meier survival curves show that mice treated with PPP or low dose of ASP3026 alone had superior overall survival compared with control mice treated with vehicle. However, mice that received a combination treatment of PPP and ASP3026 showed more improved survival than control mice. Notably, mice that received the combination treatment demonstrated statistically superior overall survival compared to mice treated with PPP or ASP3026 alone. **d** At the end of the experiments, mice were scarified and, then, tumors were collected, processed, and examined microscopically. H&E staining shows that tumors from control mice as well as tumors from mice treated with PPP or a low dose of ASP3026 alone demonstrate sheets of large anaplastic cells with numerous atypical mitotic forms. In contrast, tumors from mice treated simultaneously with PPP and a low dose of ASP3026 showed extended areas of necrosis (right side of H&E-stained section from the PPP + ASP group) in between residual tumor cells. All tumors were positive for NPM-ALK. PPP or a low dose of ASP3026 alone modestly decreased the proliferation of NPM-ALK^+^ T cell lymphoma cells, as illustrated by the Ki-67 stain. In a clear contrast, combined treatment with PPP and ASP3026 was associated with a remarkable decrease in the proliferation of these lymphoma cells with a complete absence of cell proliferation in the extended necrotic areas (right side of Ki-67-stained section from the PPP + ASP group). Similarly, whereas a slight decrease was noted in phosphorylated IGF-IR and STAT3 after treatment with PPP or a low concentration of ASP3026 alone, a pronounced reduction in the levels of these survival-promoting proteins was observed after combined treatment. Notable PPP or ASP3026 alone slightly decreased the frequency of expression of phosphorylated STAT3 in the nucleus. The effects on phosphorylated STAT3 localization in the nucleus became more evident when combined treatment with PPP and ASP3026 was employed. (Original magnification for all photomicrographs: × 400)

To further validate our in vitro results, Karpas 299 cells permanently expressing firefly luciferase were injected i.v. into C.B-17 SCID mice. Within approximately 3 weeks, an aggressive NPM-ALK^+^ T cell lymphoma was systemically disseminated in these mice. Mice were treated with a low dose of ASP3026 (5.0 mg/kg) alone (seven mice), PPP (20.0 mg/kg) alone (13 mice), or a combination of the two drugs (seven mice). In addition, a control group of 19 mice was also studied. The treatment scheme is shown in Fig. 7a. Lymphoma tumor burden was monitored weekly using bioluminescence imaging. Tumor growth was slightly decreased in mice treated with ASP3026 or PPP alone compared with vehicle-treated control mice (Fig. 7b). Notably, mice receiving the combination treatment showed substantial tumor growth suppression (Fig. 7b). In line with these observations, combining PPP and ASP3026 increased significantly mice survival compared with control mice or mice treated with PPP or ASP3026 alone (Fig. 7c). Kaplan–Meier survival curves show that mice treated with PPP or low dose of ASP3026 alone had statistically superior overall survival compared with control mice treated with vehicle (*P* < 0.05 for PPP; *P* < 0.01 for ASP3026). However, mice that received a combination treatment of PPP and ASP3026 showed more improved survival when compared with control mice (*P* < 0.0001). Furthermore, mice that received the combination treatment demonstrated statistically superior overall survival compared to mice treated with PPP or ASP3026 alone (*P* < 0.001 for PPP; *P* < 0.05 for ASP3026), (Fig. 7c). In a subgroup of mice (seven mice), a low dose of CHOP (1/3 of the standard dose; CHOP1/3) alone could not halt lymphoma tumor growth (Additional file 1: Figure S1). Nonetheless, the addition of PPP to CHOP1/3 effectively suppressed tumor growth (*P* < 0.05).

Analysis of lymphoma tumor tissues from mice showed that, compared with control mice, treatment with low concentrations of PPP and ASP3026 was associated with extended areas of tumor necrosis (Fig. 7d). A small dose of PPP or ASP3026 alone slightly decreased the proliferation of NPM-ALK^+^ T cell lymphoma cells as evident by Ki67 staining (Fig. 7d). In contrast, combining PPP and ASP3026 was associated with a remarkable decrease in the proliferative potential of these lymphoma cells. Moreover, combined treatment with PPP and ASP3026 induced marked downregulation of the phosphorylation of IGF-IR and STAT3 than treatment with one of the drugs alone (Fig. 7d).

## Discussion

In this paper, we used two clinically utilized small molecule inhibitors, PPP and ASP3026, to antagonize the oncogenic kinases IGF-IR and ALK, respectively, in NPM-ALK^+^ T cell lymphoma [24]. Simultaneous treatment with PPP and ASP3026 caused a remarkable decrease in cell viability, proliferation, and anchorage-independent colony formation. The combined treatment regimen was also associated with a marked increase in apoptotic cell death. It is important to emphasize that combined treatment with the two inhibitors induced synergistic effects and was much more pronounced than the treatment with any of these two inhibitors alone. As a possible explanation for these findings, combined treatment abolished the phosphorylation of IGF-IR and NPM-ALK as well as the phosphorylation of their common downstream target STAT3; a major survival-promoting transcription factor in this lymphoma [11, 12, 48]. When utilized together, PPP and ASP3026 increased significantly the levels of cleaved PARP, which could explain, at least in part, the remarkable increase in apoptosis when the combined regimen was used. We also found that acquired resistance to ALK inhibition could be associated with upregulation of IGF-IR phosphorylation. Of important note is that NPM-ALK^+^ T cell lymphoma cells resistant to ALK inhibition remained sensitive to IGF-IR inhibition.

In addition to the in vitro findings, similar in vivo results were observed when a low dose of ASP3026 was simultaneously used with PPP to treat systemically disseminated NPM-ALK^+^ T cell lymphoma in mice. The combination treatment regimen was not only more effective in suppressing lymphoma tumor growth, but it also improved the survival of the mice. It is important to emphasize that PPP potentiated the effects of the low dose of ASP3026 despite the fact that PPP was only used for 3 weeks of the entire 6 weeks of treatment. Histologically, combined treatment with PPP and low dose of ASP3026 was associated with expanded areas of tumor cell necrosis, decreased proliferation index as evaluated by Ki-67 staining, and downregulation of phosphorylated IGF-IR and STAT3. The effects induced by combined treatment were more pronounced than the changes noted when mice were treated by PPP or ASP3026 alone. Interestingly, treatment with PPP or ASP3026 alone slightly decreased the nuclear localization of phosphorylated STAT3, and combined treatment caused a much more pronounced decrease in such localization. These observations are consistent with our previous studies in NPM-ALK^+^ T cell lymphoma that showed inhibition of IGF-IR by PPP decreased significantly the binding of STAT3 to DNA [21].

NPM-ALK^+^ T cell lymphoma is an aggressive neoplasm. Classical treatments have primarily included combination chemotherapies with CHOP being the most commonly utilized regimen. However, previous studies demonstrated that up to 40% of the patients develop CHOP resistance and eventually relapse. Moreover, patients also die because of CHOP-related complications that are not directly related to their lymphoma [49]. We have previously noticed that the utilization of CHOP at the standard dose used in mice with subcutaneous xenografts of non-NPM-ALK^+^ T cell lymphoma [50, 51] was associated with pronounced toxicity that frequently caused early expiration of mice with systemic NPM-ALK^+^ T cell lymphoma [26]. Therefore, in the current study, we resorted to treat a subgroup of mice with only 1/3 of the standard concentration of CHOP (CHOP1/3). The growth inhibitory effects of combining PPP and CHOP1/3 in NPM-ALK^+^ T cell lymphoma were significantly more pronounced than the effects of CHOP1/3 alone.

More recently, selective ALK small molecule inhibitors have emerged as an alternative approach with enhanced effects as well as with improved safety margin [52]. Notably, the ALK small molecule inhibitors have been more evaluated in ALK^+^ solid tumors such as EML4-ALK^+^ non-small cell lung cancer. For instance, crizotinib, the prototype of first-generation ALK inhibitors, was primarily used to treat non-small cell lung cancer patients with ALK expression in their tumors. Although crizotinib initially demonstrated promising effects, subsequent studies highlighted the development of significant resistance as a major hurdle to crizotinib [53]. In a previous study, we systematically characterized the in vitro effects of ASP3026, an orally available second-generation ALK inhibitor, in NPM-ALK^+^ T cell lymphoma cell lines [26]. We also examined the effects of ASP3026 compared with CHOP in our systemic NPM-ALK^+^ T cell lymphoma model in mice. ASP3026 demonstrated remarkable in vitro and in vivo effects and was superior to and safer than CHOP. Moreover, ASP3026 successfully overcame the resistance to crizotinib. However, subsequent studies demonstrated that resistance also develops to ASP3026 because of ALK mutations [27]. In the current study, we examined the effects of a much lower concentration of ASP3026 than the one we used previously (5 mg/kg/day vs. 30 mg/kg/day) [26]. Nonetheless, combining PPP with this very low concentration of ASP3026 caused pronounced in vivo inhibitory effects in NPM-ALK^+^ T cell lymphoma tumors and improved mice survival as well. These pronounced effects occurred despite the fact that PPP was administered for only 3 weeks out of the entire 6 weeks of treatment.

IGF-IR is a receptor tyrosine kinase with potent oncogenic potential that has been observed in numerous types of cancer including hematological neoplasms [54]. We have previously demonstrated that IGF-IR is highly expressed in NPM-ALK^+^ T cell lymphoma [21]. Increased expression of IGF-IR in this lymphoma can be attributed, at least in part, to decreased expression of the transcription factors Ikaros isoform 1 (Ik-1) and myeloid zinc finger 1 (MZF1) [55]. Under the physiological conditions, Ik-1 and MZF1 appear to repress the expression of the *IGF*-*IR* gene in normal T lymphocytes. Moreover, we have provided evidence to support that IGF-IR and NPM-ALK are physically associated and interact reciprocally to enhance their phosphorylation/activation as well as the activation of their common downstream modulators including STAT3 [21, 22]. Furthermore, it appears that the association between IGF-IR and NPM-ALK plays critical roles in maintaining the stability of NPM-ALK protein [22]. In further support of an important role of IGF-IR in the pathogenesis of NPM-ALK^+^ T cell lymphoma, our current data show that acquired resistance to ALK inhibition is associated with a remarkable increase in the phosphorylation of IGF-IR. The increase in IGF-IR phosphorylation is an event that probably occurred secondary to the increase in the phosphorylation of NPM-ALK, which was noted in cells resistant to ALK inhibition. Nonetheless, our data show that inhibition of IGF-IR remained to be substantially effective in decreasing the viability of NPM-ALK^+^ T cell lymphoma cells resistant to ALK inhibition. Most likely, high levels of phosphorylation of IGF-IR detected in these cells enhanced their dependence on IGF-IR signaling and, as a result, maintained their sensitivity to IGF-IR inhibition. Our results show that treatment of the NPM-ALK^+^ T cell lymphoma cell line DEL-R, which was resistant to the ALK inhibitor, with PPP induced downregulation of pIGF-IR and pNPM-ALK. These results are in agreement with our previous findings demonstrating the association and reciprocal interactions through phosphorylation that exist between IGF-IR and NPM-ALK in this lymphoma [21, 22]. Notably, however, PPP did not only abrogate the phosphorylation of IGF-IR and NPM-ALK in the ALK inhibitor-resistant cells but also downregulated the basal levels of these two oncogenic kinases. Several previous studies have demonstrated similar effects of PPP on the basal levels of IGF-IR [56–58]. The effects of PPP might be attributed to the fact that it, similar to other small molecule kinase inhibitors, induces selective but not entirely specific effects. For instance, one possible mechanism for PPP effects on the basal levels of IGF-IR could be attributed to the MDM2 E2 ligase-induced ubiquitination and subsequent downregulation of IGF-IR expression after treatment with PPP [56]. Similar to our findings in the ALK-inhibitor-resistant cells, PPP-induced downregulation of IGF-IR was shown to be pronounced in multidrug resistant cancer cells [58]. On the other hand, we have previously found that the association between IGF-IR and NPM-ALK through Tyr^644^ and Tyr^664^, located within the C terminus of NPM-ALK, is required to maintain the stability of NPM-ALK protein [22]. Therefore, the decrease in the basal levels of IGF-IR after treating the ALK inhibitor-resistant cells with PPP could explain the simultaneous decrease in NPM-ALK basal protein levels. Although, future studies are required to further analyze these important observations, our data support that strategies based on exploiting IGF-IR signaling might represent a legitimate approach to overcome ALK resistance.

One of the strategies that has been suggested in previous preclinical studies in epithelial tumors to enhance the effects of ALK inhibitors as well as to avoid the resistance to these inhibitors is to combine ALK inhibition with IGF-IR inhibition [59–61]. To our knowledge, such an approach has never been examined in NPM-ALK^+^ T cell lymphoma, the prototype of ALK^+^ malignant neoplasms. Moreover, previous studies in NPM-ALK^+^ T cell lymphoma as well as ALK^+^ lung cancer have shown cross-resistance among various ALK inhibitors [28, 62, 63]. Combined treatment with low doses of ALK inhibitors in combination with IGF-IR inhibitors might bypass cross-resistance. Our results strongly suggest that combining low doses of ALK inhibitors with IGF-IR inhibitors might represent an effective strategy to successfully eradicate this aggressive lymphoma. Importantly, the utilization of such low concentrations of ALK might help to avoid development of therapeutic resistance as well as to increase the clinical tolerance to these therapeutic agents when used alone at higher concentrations.

## Conclusions

We present substantial in vitro and in vivo evidence to support that simultaneous utilization of PPP and low concentrations of ASP3026 to antagonize the activity of IGF-IR and NPM-ALK, respectively, could represent a superior strategy to treat the aggressive NPM-ALK^+^ T cell lymphoma than using higher concentrations of each of these two drugs alone. Our data also show that acquired resistance to ALK inhibition could be associated with substantial increase in the phosphorylation/activation of IGF-IR, which implies that the utilization of IGF-IR inhibitors might be a reasonable strategy to overcome resistances to ALK inhibitors. The findings presented herein are relevant not only to NPM-ALK^+^ T cell lymphoma but also to other types of cancer that simultaneously express ALK and IGF-IR. Performing clinical studies to further support our findings is feasible because of the availability of IGF-IR and ALK inhibitors that have been already approved to be used in patients with different types of cancer.

## Additional file

Additional file 1: **Figure S1.** Combined treatment with a low dose of CHOP and PPP suppresses NPM-ALK^+^ ALCL cell tumor growth *in vivo.* (A) Mice developed NPM-ALK^+^ ALCL tumors within approximately 3 weeks after injection, and were monitored weekly using bioluminescence imaging. The intensity of the signal is indicated by color (blue: low; green: intermediate; red: high tumor burden). Aggressive lymphoma growth was observed in mice treated with a low dose of CHOP (CHOP1/3). However, mice receiving CHOP1/3 in addition to PPP (20 mg/kg) showed almost complete suppression of tumor growth at 4 weeks after injection. (B) Kaplan-Meier survival curves show that mice treated with chop1/3 and PPP (20 mg/kg) demonstrated statistically superior overall survival compared to mice treated with CHOP1/3 alone (*P*<0.05). (TIF 312 kb)

## Data Availability

All data generated or analyzed during this study are included in this published article and its supplementary information file.

## Abbreviations

ANOVA: Analysis of variance
CHOP: Cyclophosphamide, doxorubicin, vincristine, and prednisone
DMSO: Dimethylsulfoxide
EDTA: Ethylenediaminetetraacetic acid
ERK: Extracellular signal-regulated kinase
FITC: Fluorescein isothiocyanate
GFP: Green fluorescent protein
hLUC: Humanized firefly luciferase
i.p.: Intraperitoneal
i.v.: Intravenous
IGF-IR: Type I insulin-like growth factor receptor
Ik-1: Ikaros isoform 1
JAK: Janus kinase
MAPK: Mitogen-activated protein kinase
MZF1: Myeloid zinc finger 1
NPM-ALK: Nucleophosmin-anaplastic lymphoma kinase
PARP: Poly ADP ribose polymerase
PI: Propidium iodide
PI-3K: Phosphatidylinositol 3 kinase
PPP: 1picropodophyllin
RPMI: Roswell Park Memorial Institute
s.c.: Subcutaneous
SCID: Severe combined immunodeficiency
STAT: Signal transducer and activator of transcription
