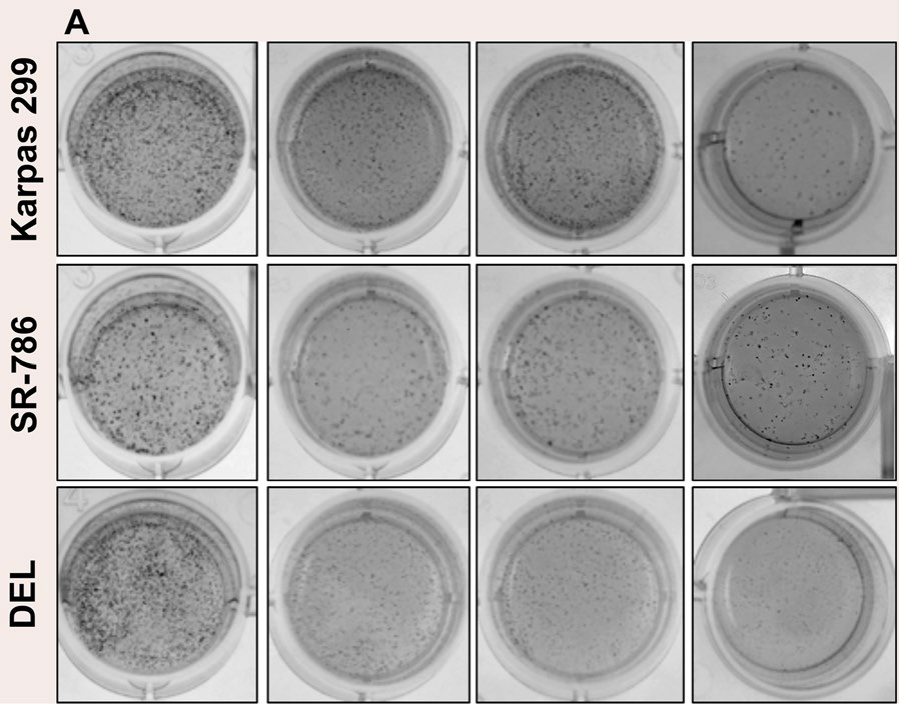# Correction: Dual inhibition of IGF-IR and ALK as an effective strategy to eradicate NPM-ALK^+^ T-cell lymphoma

**DOI:** 10.1186/s13045-023-01458-w

**Published:** 2023-06-13

**Authors:** Bhawana George, Suraj Konnath George, Wenyu Shi, Abedul Haque, Ping Shi, Ghazaleh Eskandari, Magnus Axelson, Olle Larsson, Ahmed O. Kaseb, Hesham M. Amin

**Affiliations:** 1 Department of Hematopathology, The University of Texas MD Anderson Cancer Center, Unit 072, 1515 Holcombe Boulevard, Houston, TX 77030 USA; 2 Department of Hematology, Affiliated Hospital of the University of Nantong, Jiangsu, China; 3 State Key Laboratory of Bioreactor Engineering, East China University of Science and Technology, Shanghai, China; 4 Department of Molecular Medicine and Surgery, Karolinska Institute, Stockholm, Sweden; 5 Department of Oncology and Pathology, Karolinska Institute, Stockholm, Sweden; 6 Depertment of Gastrointestinal Medical Oncology, The University of Texas MD Anderson Cancer Center, Houston, TX USA; 7 MD Anderson Cancer Center UTHealth Graduate School of Biomedical Sciences, Houston, TX USA

**Correction: Journal of Hematology & Oncology (2019) 12:80** **https://doi.org/10.1186/s13045-019-0768-8**

The original article [1] contains an error in Fig. 4a whereby the figure of one well that belonged to one cell line was used in another cell line.

The corrected image can be viewed ahead.