# Supplementary figures and images for: Dual inhibition of IGF-IR and ALK as an effective strategy to eradicate NPM-ALK+ T-cell lymphoma
Source: J Hematol Oncol. 2019 Jul 24;12:80. doi: 10.1186/s13045-019-0768-8 (PMC6657048; doi:10.1186/s13045-019-0768-8)

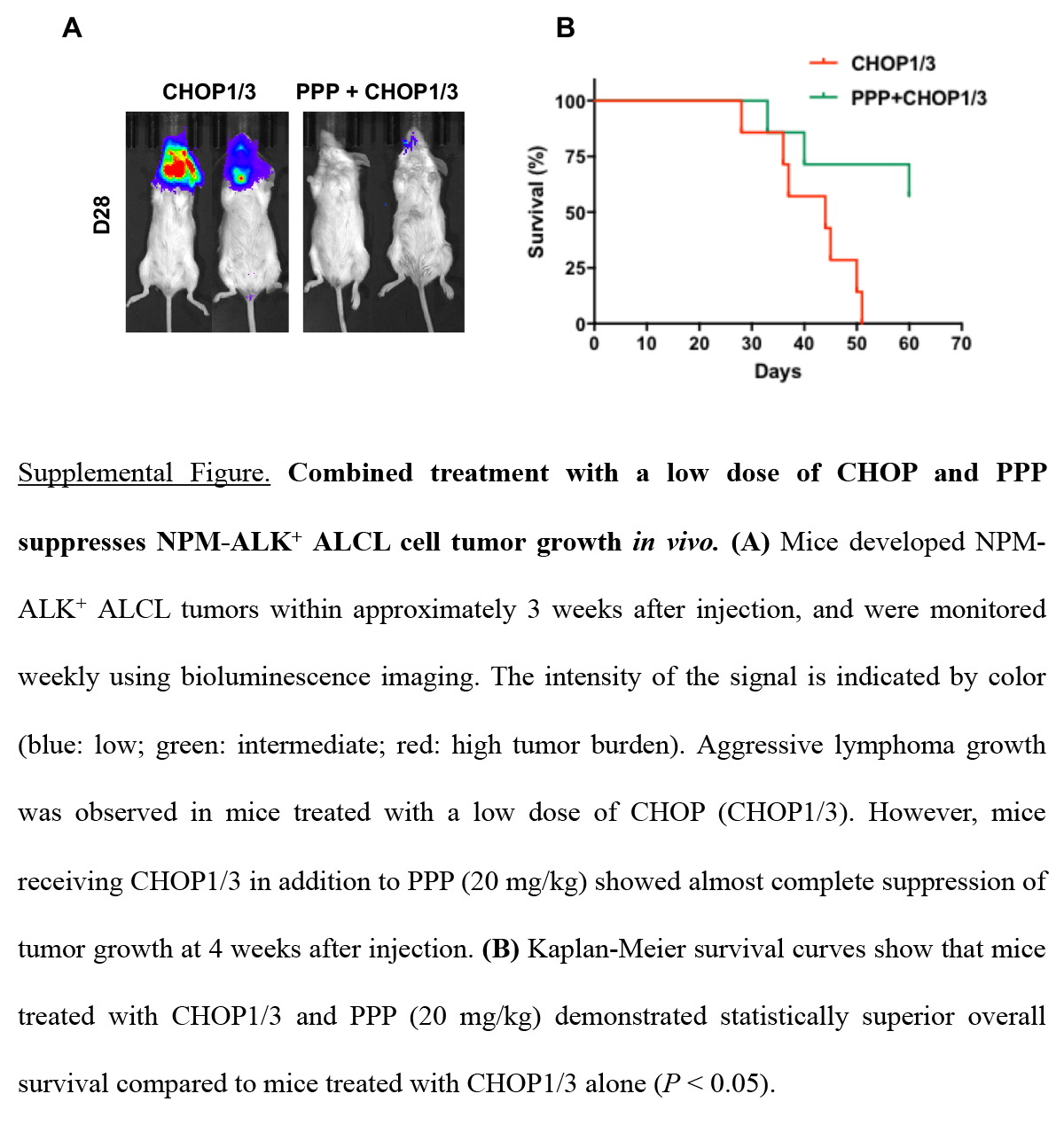

Supplement: Supplementary file 1 — Figure S1. Combined treatment with a low dose of CHOP and PPP suppresses NPM-ALK+ ALCL cell tumor growth in vivo. (A) Mice developed NPM-ALK+ ALCL tumors within approximately 3 weeks after injection, and were monitored weekly using bioluminescence imaging. The intensity of the signal is indicated by color (blue: low; green: intermediate; red: high tumor burden). Aggressive lymphoma growth was observed in mice treated with a low dose of CHOP (CHOP1/3). However, mice receiving CHOP1/3 in addition to PPP (20 mg/kg) showed almost complete suppression of tumor growth at 4 weeks after injection. (B) Kaplan-Meier survival curves show that mice treated with chop1/3 and PPP (20 mg/kg) demonstrated statistically superior overall survival compared to mice treated with CHOP1/3 alone (P<0.05). (TIF 312 kb) [file 13045_2019_768_MOESM1_ESM.tif]
